# Who Likes Extraverts? Testing the Interplay Between Perceiver Needs and Target Appearance in Impression Formation

**DOI:** 10.5334/irsp.996

**Published:** 2026-03-26

**Authors:** Bastian Jaeger, Alex L. Jones, Liam Satchell, Christoph Schild, Florian van Leeuwen

**Affiliations:** 1 Department of Social Psychology, Tilburg University, Tilburg, The Netherlands; 2 Department of Psychology, Swansea University, Swansea, United Kingdom; 3 School of Psychology, Sport, and Health Sciences, University of Portsmouth, United Kingdom; 4 Research and Innovation, University of Winchester, United Kingdom; 5 Department of Psychology, University of Münster, Münster, Germany

**Keywords:** impression formation, social perception, needs, motives, extraversion

## Abstract

The role of perceiver differences in impression formation remains relatively poorly understood. One line of research has tried to understand these differences by exploring the role of perceivers’ needs and motivations, reasoning that perceivers should form more positive impressions of targets who appear more likely to address their needs. For example, a perceiver with a stronger affiliation motive might have a more positive impression of someone who looks more (vs. less) extraverted. We conducted two preregistered replication studies of proposed associations between three individual difference variables and likeability impressions of individuals varying in perceived extraversion. Using the original stimuli and study design (Study 1, *n* = 273) and two additional stimulus sets and an improved study design (Study 2, *n* = 367), we did not find that individual differences in (a) affiliative needs, (b) pathogen concern, or (c) sociosexual orientation were associated with likeability impressions of individuals varying in perceived extraversion. Bayesian analyses supported this conclusion. Our findings highlight the need for additional research to understand how individual differences shape social perception.

Many studies have examined associations between specific facial cues and first impressions, but recent work has highlighted that there are also substantial individual differences in impression formation (Hehman et al. 2019). Thus, to understand how first impressions are formed, researchers need to examine the interplay between characteristics of the person being judged *and* the person forming the judgment. Despite the apparent importance of target × perceiver interactions in social perception, previous studies have almost exclusively focused on the role of target characteristics (Hehman et al. 2019). The question of how individual differences shape first impressions has remained relatively unexplored. Perhaps the most extensive set of studies on how target and perceiver characteristics interact to shape social perception was conducted by Brown and Sacco (2016a; 2016b; 2017) who investigated whether various needs and motivations of perceivers predict their impressions of targets varying in apparent extraversion. Given the theoretical importance of individual differences in social perception and the relative paucity of empirical studies on this topic thus far, we aimed to replicate and extend three key findings from this line of work.

## Perceiver Needs and Apparent Extraversion

One fundamental human motive is to socialize and form lasting relationships with others—often referred to as the ‘need to belong’ (Baumeister & Leary 1995). A stronger need to belong is associated with a stronger motivation to seek out contact with others and with more negative reactions to social exclusion (Leary et al. 2013). People with a stronger need to belong should be more motivated to seek out individuals who can fulfill their needs for social connection. Extraverted individuals are more gregarious and have larger social networks (Leary et al. 2013), making them more desirable interaction partners for people with a stronger need to belong. Although people might not know how extraverted strangers are, research suggests that people readily infer this based on others’ facial appearance (Oosterhof & Todorov 2008). In line with this reasoning, one study showed a positive association between perceivers’ need to belong and their preferences for extraverted-looking (vs. introverted-looking) individuals (Brown & Sacco 2017). When participants viewed pairs of images of the same targets—one version manipulated to appear relatively extraverted, the other version manipulated to appear relatively introverted—those with a stronger need to belong more often selected the extraverted-looking image as their preferred appearance. That is, people with a stronger need to belong formed more positive impressions of individuals who *look* extraverted.

A similar argument was made to explain why individual differences in pathogen concern are negatively related to extraversion preferences, a finding that emerged in another study (Brown & Sacco 2016a). Infectious disease has been a recurrent threat in humans’ evolutionary past and interpersonal contact can increase an individual’s risk of pathogen exposure (Schaller & Park 2011). As extraverted individuals are a more likely source of infectious disease due to their increased social activity and larger social networks (Leary et al. 2013), people who are chronically concerned about pathogen threats may avoid extraverted-looking others.

Another study found that a less restricted sociosexual orientation—an increased motivation for casual sex and shorter-term sexual partners (Penke & Asendorpf 2008)—was positively related to facial extraversion preferences (Brown & Sacco 2016b). As extraverted individuals are more open to sexual relationships (Nettle 2005), they may seem more approachable to people with a less restricted sociosexual orientation. Together, these findings provide initial evidence on the interplay between perceiver characteristics (e.g., pathogen concern) and target characteristics (e.g., perceived extraversion) in impression formation (here, first impressions of likeability).

## A Critical Examination of the Empirical Evidence

Although the series of results described shows how chronically activated motives can explain individual differences in (one domain of) impression formation, the available evidence provides only weak and mixed support for the hypotheses. In the study that examined the role of individual differences in pathogen concern in impression formation (Brown & Sacco 2016a), pathogen concern was measured with the Perceived Vulnerability to Disease (PVD) scale (Duncan et al. 2009). Although the Germ Aversion subscale better captures individual differences in pathogen concern (Duncan et al. 2009), an association with extraversion preferences was surprisingly only observed for the Perceived Infectability subscale (and this association was only ‘marginally significant’).^1^ Results for the role of individual differences in sociosexual orientation were also mixed. While stronger preferences for extraverted-looking individuals were hypothesized to emerge particularly among female perceivers judging male targets,^2^ this prediction was not borne out by the data. A positive correlation between sociosexual orientation and extraversion preferences was observed for male (but not female) targets, but this association did not differ between male and female participants (Brown & Sacco 2016b).

The present work focuses on chronically activated motives in impression formation given recent work suggesting that individual differences between perceivers account for a substantial amount of variation in impression formation. However, motives can also be experimentally activated to test their effect on impression formation. In two separate studies, making disease threats salient did not affect participants extraversion preferences (Brown, Medlin et al. 2019; Brown & Sacco 2016a). Moreover, socially excluding participants, which increased their need for affiliation, did increase their extraversion preferences, but this effect surprisingly only emerged for male participants (Brown, Sacco et al. 2019). Although these results do not speak directly to the role of individual differences in pathogen avoidance motivations and the need to belong (*state* effects and *trait* effects do not necessarily converge), they cast some additional doubt on the robustness of the original findings.

In all three studies that examined the role of trait effects, impressions of targets varying in perceived extraversion were measured with a two-alternative forced-choice (2AFC) design (Brown & Sacco 2016b; Brown & Sacco 2016a; Brown & Sacco 2017). Participants viewed 40 pairs of face images and each pair contained two images of the same target that was manipulated to vary in perceived extraversion. This method has been criticized for its poor external validity—people do not encounter twin faces that only vary on one dimension in everyday life—and for its proneness for producing false positive inferences (Bovet et al. 2022; DeBruine 2020). The 2AFC design highlights even subtle differences between face pairs and participants may rely on these differences even though these would go unused (or even unnoticed) in everyday life. In other words, it is plausible that resulting associations between a cue and a judgment are an artifact of the study design, rather than being reflective of which cues participants would *spontaneously* rely on under natural conditions.

The work by Brown and Sacco (2016a; 2016b; 2017), which we refer to as ‘the original studies’ below, offers concrete, theory-driven predictions on how target and perceiver characteristics might interact in impression formation, an area that has remained relatively unexplored. Yet, due to the mixed results and the fact that all studies used a methodology that is prone to produce false positive inferences, additional, stronger tests of the hypotheses are needed.

## The Present Studies

Here, we present the results of two preregistered replication and extension studies, in which we examine the role of three individual difference variables in social perception. Specifically, in both studies, we test whether perceivers form more positive impressions of extraverted-looking (vs. introverted-looking) individuals if they score higher on the need to belong (Brown & Sacco 2017), lower on pathogen concern (Brown & Sacco 2016a), and higher on an unrestricted sociosexual orientation (this association was expected to emerge specifically among female perceivers rating male targets; Brown & Sacco 2016b). We test these hypotheses with two independent samples of participants and three samples of face stimuli, moving from a relatively close replication in Study 1 (*n* = 273) to a more conceptual replication that attempts to improve on the methodology of the original studies in Study 2 (*n* = 367; LeBel et al. 2019; see the Supplemental Materials for a detailed overview of differences between the original studies and our replication studies). In both studies, we report the results of both frequentist and Bayesian analyses (Wagenmakers 2007).

## Study 1

In Study 1, we used the same set of 40 face pairs and the same rating technique (2AFC) that were used in the original studies. We examined whether participants who report lower pathogen concern, a stronger need to belong, and a less restricted sociosexual orientation would form more positive impressions of extraverted-looking targets. The association with sociosexual orientation was expected to emerge specifically among female perceivers rating male targets (Brown & Sacco 2016b).^3^

### Methods

#### Participants

The study was advertised in various Facebook groups of Dutch universities and participants took part voluntarily. In line with our preregistered exclusion criteria data from 4 participants (1.44%) who indicated poor English proficiency were excluded, leaving a final sample of 273 participants (*M_age_* = 23.18 years, *SD_age_* = 6.14; 69.60% female, 29.67% male, 0.73% other). A sensitivity analysis showed that with our final sample size, we were able to detect correlations as small as *r* = .17 with 80% power (and *α* = 5%). Our study had 94% power to detect the effect size that was reported for sociosexual orientation in the original study (*r* = .21; Brown & Sacco 2016b), but only 76% power to detect the reported effect size for the need to belong (*r* = .16; Brown & Sacco 2017). For pathogen concern, the relevant effect sizes were not reported (Brown & Sacco 2016a). Note that these are only conservative approximations as our multilevel regression analyses should be more powerful in detecting the hypothesized associations (Baayen et al. 2008). More details on our sample size planning and on differences between the original and the present studies are reported in the Supplemental Materials.

#### Materials and Procedure

We used the same stimuli and rating technique as in the original studies (Brown & Sacco 2016b; Brown & Sacco 2016a; Brown & Sacco 2017). Participants viewed 40 pairs of facial photographs. Each pair of photographs showed the face of the same individual. Perceived extraversion was manipulated by morphing the face with prototypically introverted- and extraverted-looking faces (Holtzman 2011). Each face represented a 50%/50% morph of the original face and the prototype. Thus, each pair contained an introverted and an extraverted version of the same face and participants were asked to indicate which version they ‘prefer’ in a two-alternative forced-choice design. We randomized the order in which the face pairs were displayed to participants. On each trial, we also randomized whether the introverted- or extraverted-looking face was presented on the left or right.

Next, participants completed various questionnaires (see Tables S1 and S2 and Figure S1 in the Supplemental Materials for descriptive statistics). We measured individual differences in pathogen concern with the 15-item Perceived Vulnerability to Disease Scale (Duncan et al. 2009). The Perceived Infectability subscale (*α* = 0.86) captures beliefs about a person’s subjective susceptibility to contagious disease. The Germ Aversion subscale (*α* = 0.70) captures differences in affective responses to pathogen threats. Participants indicated their responses on a 7-point scale ranging from 1 (*strongly disagree*) to 7 (*strongly agree*).

We measured individual differences in affiliative needs with the 10-item Need to Belong scale (Leary et al. 2013; *α* = 0.75). Participants indicated their responses on a 5-point scale ranging from 1 (*not at all*) to 5 (*extremely*).

We measured individual differences in sociosexual orientation with the 9-item Sociosexual Orientation Inventory-Revised (Penke & Asendorpf 2008), which captures preferences for uncommitted vs. committed sexual relationships.^4^ The scale consists of three subscales, which measure sexual behavior, attitudes towards casual sex, and sexual desire. Participants indicated their responses on 9-point scales, with higher scores indicating a less restricted sociosexuality. In line with the original study (Brown & Sacco 2016b), we focus on participants’ average score across the three subscales (*α* = 0.83).

#### Analysis strategy

Multilevel regression models were estimated in R using the *lme4* and *lmerTest* packages. We also conducted Bayesian analyses following the approach outlined in Wagenmakers (2007; see Supplemental Materials for more details). For interpretative convenience, we always display Bayes factors so that they reflect support for the favored hypothesis (i.e., *BF_10_* when evidence favors the alternative hypothesis and *BF_01_* when evidence favors the null hypothesis). In line with Jeffreys (1961), we label evidence as anecdotal (1 ≤ *BF* <3), substantial (3 ≤ *BF* <10), strong (10 ≤ *BF* <30), very strong (30 ≤ *BF* <100), or decisive (*BF* ≥100). In Table S3 of the Supplemental Materials, we also present correlation coefficients between the individual difference variables and participants’ extraversion preferences (mirroring the analysis strategy in the original studies).

### Results

**Figure 1:**
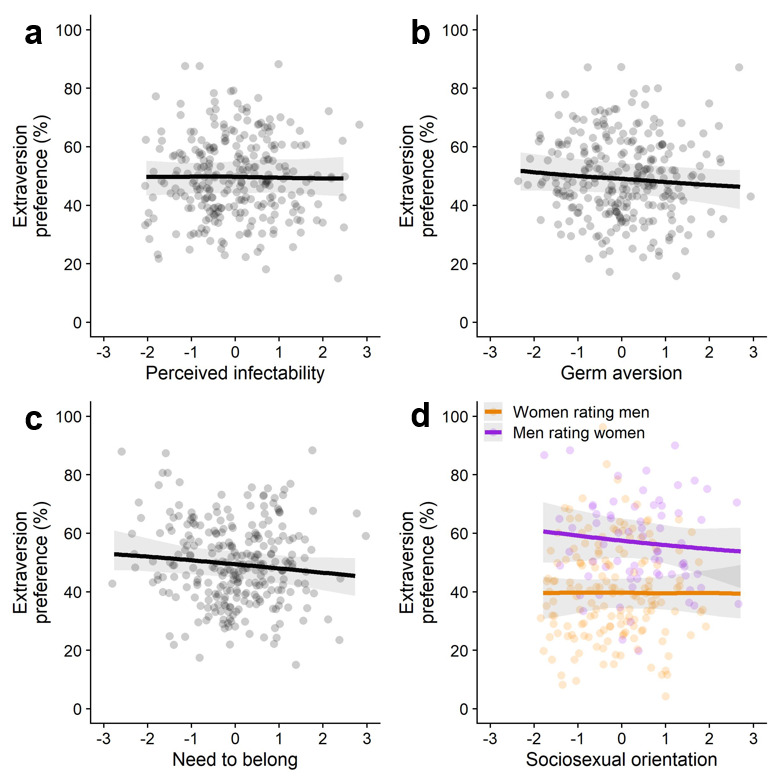
Predicted relations between facial extraversion preferences and individual differences in **(a)** perceived infectability, **(b)** germ aversion, **(c)** need to belong, and **(d)** sociosexual orientation (the graph displays results for the predicted interaction effect with participants’ gender; Study 1). *Note*. All individual difference measures were z-standardized.

We coded participants’ responses as 0 when they preferred the introverted-looking face and as 1 when they preferred the extraverted-looking face. Next, we conducted our critical tests. We examined the associations between facial extraversion preferences and four individual difference measures. Four separate multilevel logistic regression models with random intercepts per participant and target were estimated in which face preference (0 = introverted-looking, 1 = extraverted-looking) was regressed on the individual difference measures (results for all models are displayed in Figure 1).

First, we examined whether participants scoring higher on pathogen concern showed stronger extraversion preferences. There were no significant associations between extraversion preferences and perceived infectability (with decisive evidence in favor of the null hypothesis), *b* = –0.003, *SE* = 0.04, *OR* = 1.00, 95% CI [0.92, 1.07], *p* = .94, *BF_01_* = 104.2 (see Figure 1a), or germ aversion (with very strong evidence in favor of the null hypothesis), *b* = –0.04, *SE* = 0.04, *OR* = 0.96, 95% CI [0.89, 1.04], *p* = .33, *BF_01_* = 64.61 (see Figure 1b). We also tested whether a significant association for perceived infectability would emerge when restricting our analysis to male perceivers rating female targets (as was reported in Brown & Sacco 2016a), but we did not find support for this either (see Supplemental Materials).

We also found no significant association between extraversion preferences and individual differences in need to belong (with very strong evidence in favor of the null hypothesis), *b* = –0.06, *SE* = 0.04, *OR* = 0.94, 95% CI [0.88, 1.02], *p* = .13, *BF_01_* = 33.10 (see Figure 1c).

In the original study, it was predicted that people with a less restricted sociosexual orientation would show stronger extraversion preferences and that this association would be stronger for women rating men (vs. men rating women; Brown & Sacco 2016b). We therefore estimated a model in which face preference was regressed on sociosexual orientation (with higher scores indicating a less restricted sociosexual orientation), gender (–0.5 = women rating men, 0.5 = men rating women), and their interaction. There was a significant effect of gender (with strong evidence in favor of the alternative hypothesis), *b* = 0.74, *SE* = 0.18, *OR* = 2.10, 95% CI [1.46, 3.03], *p* < .001, *BF_10_* = 21.94, showing that men (rating women) showed stronger extraversion preferences than women (rating men). We did not find an effect of sociosexual orientation (with very strong evidence in favor of the null hypothesis), *b* = –0.04, *SE* = 0.06, *OR* = 0.96, 95% CI [0.86, 1.07], *p* = .50, *BF_01_* = 54.10, and the predicted interaction effect was also not significant (with very strong evidence in favor of the null hypothesis), *b* = –0.07, *SE* = 0.11, *OR* = 0.94, 95% CI [0.75, 1.16], *p* = .56, *BF_01_* = 57.19 (see Figure 1d).

### Discussion

In Study 1, we conducted a relatively close replication of three studies that examined the role of individual differences in social perception (Brown & Sacco 2016b; Brown & Sacco 2016a; Brown & Sacco 2017). Using the same face stimuli and the same study design, we tested whether participants who score lower on pathogen concern, participants who score higher on need to belong, and participants (particularly female perceivers rating male targets) who report a less restricted sociosexual orientation show a stronger preference for extraverted-looking others. We did not find support for any of the three hypotheses and Bayesian analyses showed strong to decisive evidence in favor of the null hypothesis (see Table 1 for an overview).

**Table 1:** Overview of the key hypotheses and results across the two studies.


	STUDY 1 (ORIGINAL STIMULI)		STUDY 2 (BASEL FACE STIMULI)		STUDY 2 (10K FACE STIMULI)	
		
	FREQUENTIST	BAYESIAN	FREQUENTIST	BAYESIAN	FREQUENTIST	BAYESIAN

Perceived infectability (–)	×	×	✓	×	×	×

Germ aversion (–)	×	×	×	×	×	×

Need to belong (+)	×	×	×	×	✓	×

Sociosexual orientation: f rating m (+)^a^	×	×	×	×	✓	×

Stimulus set	Same		Different		Different	

Stimulus presentation	Same		Same		Different	


*Note*. Variables hypothesized to correlate with preferences for extraverted-looking targets are shown in column 1 (the predicted sign is indicated in parentheses). For the frequentist and Bayesian analyses, we highlight a prediction as supported if the observed association was in the predicted direction and associated with *p* < .05 and *BF* ≥3 (in favor of the alternative hypothesis), respectively. We did not specify these cut-offs in our preregistration. Adopting alternative, justifiable cut-offs for the Bayesian analyses, such as *BF* ≥10 in favor of the alternative hypothesis, would yield the same results. ^a^Following the hypotheses described in the original study, we coded this hypothesis as supported if there was a positive association for female perceivers rating male targets and if this association was stronger than the association for male perceivers rating female targets.

## Study 2

In Study 2, we conducted two additional replication attempts. We tested the same three hypotheses but measured facial extraversion preferences in two alternative ways. First, we employed the same rating method as in Study 1 (and the original studies), but we used a different set of face stimuli taken from the Basel Face Database (Walker et al. 2018). Second, given recent critiques of the 2AFC method (DeBruine 2020), we used an alternative rating method and a third set of face stimuli from the 10k Faces Database (Bainbridge et al. 2013).

### Methods

#### Participants

We recruited 390 first-year psychology students from a Dutch university who completed the study in return for partial course credit. In line with our preregistered exclusion criteria, data from 5 participants (1.28%) who indicated poor English proficiency and from 18 participants (4.68%) who completed the study on a cell phone were excluded, leaving a final sample of 367 participants (*M_age_* = 19.90 years, *SD_age_* = 3.70; 83.11% female, 15.26% male, 1.63% other). A sensitivity analysis showed that with our final sample size of 367 participants, we were able to detect correlations as small as *r* = .19 with 95% power (and *α* = 5%). Our study had 98% power to detect the effect size that was reported for sociosexual orientation in the original study (*r* = .21; Brown & Sacco 2016b), and 87% power to detect the reported effect size for the need to belong (*r* = .16; Brown & Sacco 2017). For pathogen concern, the relevant effect sizes were not reported (Brown & Sacco 2016a). Note that these are only conservative approximations as our multilevel regression analyses should be more powerful in detecting the hypothesized associations (Baayen et al. 2008). More details on our sample size planning and on differences between the original and the present studies are reported in the Supplemental Materials.

#### Materials and Procedure

Participants completed two rating tasks that measured their preferences for extraverted-looking others. First, participants viewed 40 pairs of facial photographs taken from the Basel Face Database (Walker et al. 2018). Each pair showed the face of the same individual. Perceived extraversion was manipulated by morphing the face with prototypically introverted and extraverted faces. Thus, each pair contained an introverted and extraverted face version, and participants were asked to indicate which face version they ‘prefer’ in a two-alternative forced-choice design (in line with the original studies; e.g., Brown & Sacco 2017). We randomized the order in which the face pairs were displayed and whether the introverted- or extraverted-looking face was presented on the left or right.

To reach the minimum time required for a study participation session by our department, participants then completed an unrelated study (measuring beliefs about the moral status and mental capacities of different farm animals) for approximately 3 minutes before commencing with the second rating task. Participants viewed 100 faces, which were randomly sampled from the 10k Faces Database (Bainbridge et al. 2013). The database includes ratings on a wide range of dimensions, including sociability and introversion. These ratings were provided by 30 workers recruited from Amazon Mechanical Turk who rated each face on a 9-point scale. Individual ratings were then averaged to create an average score for each face. We reverse-scored introversion ratings and averaged them with sociability ratings to create a measure of perceived extraversion (the correlation between ratings on the two dimensions was *r* = .6). Photographs were presented sequentially in a random order and participants indicated, on a 9-point scale, how much they think they would like the person in photo on a scale that ranged from 1 (*not at all*) to 9 (*extremely*).

We examined associations between facial extraversion preferences and four individual difference measures (see Tables S1 and S2 and Figure S2 in the Supplemental Materials for descriptive statistics): perceived infectability (*α* = 0.87) and germ aversion (*α* = 0.74; Duncan et al. 2009), need to belong (*α* = 0.75; Leary et al. 2013), and sociosexuality (*α* = 0.84; Penke & Asendorpf 2008),^5^ which were measured with the same scales as in Study 1.

We also explored the role of another trait in explaining facial extraversion preferences: perceivers’ extraversion. People generally have favorable impressions of others with a similar personality (Youyou et al. 2017). Thus, extraverted people may show an increased preference for extraverted-looking others. An initial study did not find support for this prediction (Sacco & Brown 2018). In the present study, we conducted an additional test of the hypothesis. We measured individual differences in extraversion with the 10-item Extraversion subscale of the HEXACO inventory (*α* = 0.82; Ashton & Lee 2009). Participants indicated their responses on a 5-point scale ranging from 1 (*strongly disagree*) to 5 (*strongly agree*). We report these results in the Supplemental Materials.

#### Analysis strategy

We followed the same analysis strategy as in Study 1. For all primary tests, we report the results of frequentist and Bayesian analyses. Correlation coefficients between the individual difference variables and participants’ extraversion preferences (mirroring the analysis strategy in the original studies) are reported in Table S3 of the Supplemental Materials.

### Results

#### 2AFC Design (Basel Faces)

**Figure 2:**
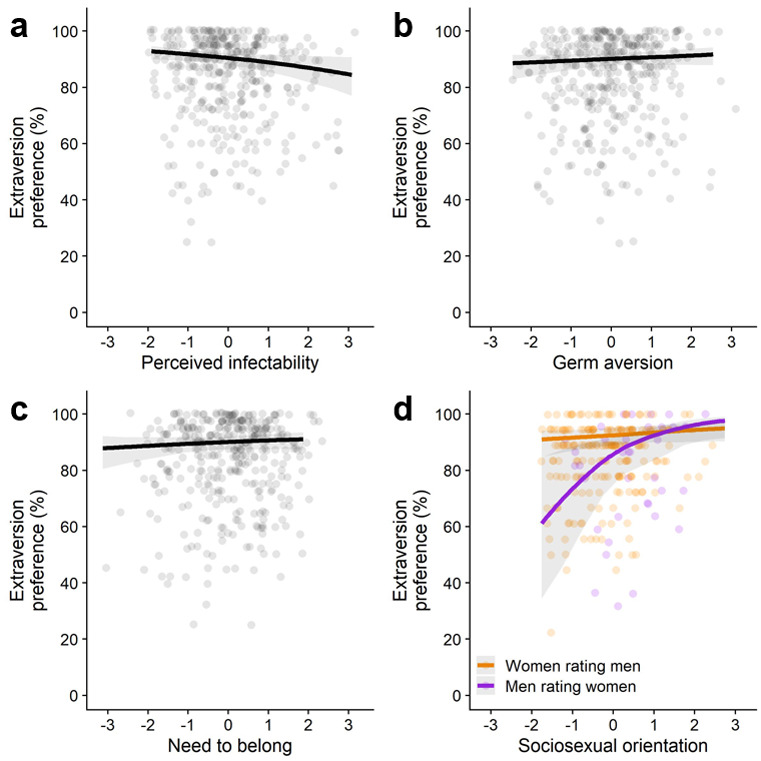
Predicted relation between facial extraversion preferences and individual differences in **(a)** perceived infectability, **(b)** germ aversion, **(c)** need to belong, **(d)** sociosexual orientation (the graph displays results for the predicted interaction effect with participants’ gender; Study 2). *Note*. All individual difference measures were z-standardized.

First, we analyzed participants’ responses when rating the face stimuli from the Basel Face Database. We coded participants’ responses as 0 when they preferred the introverted-looking face and as 1 when they preferred the extraverted-looking face. We examined associations between facial extraversion preferences and the four individual difference measures. Four separate multilevel logistic regression models with separate random intercepts per participant and target were estimated in which face preference (0 = introverted-looking, 1 = extraverted-looking) was regressed on each individual difference measure (results for all models are displayed in Figure 2).

First, we examined whether participants scoring higher on pathogen concern showed weaker extraversion preferences. There was a significant negative association between extraversion preferences and perceived infectability, *b* = –0.18, *SE* = 0.07, *OR* = 0.84, 95% CI [0.71, 0.99], *p* = .02, *BF_01_* = 7.70 (see Figure 2a). Preferences for extraverted-looking individuals were weaker among people who scored higher on perceived infectability, but this effect was very small. A one standard deviation increase in perceived infectability was associated with a 1.47 percentage point decrease in extraversion preferences and a Bayesian analysis indicated substantial evidence in favor of the null hypothesis. We also tested whether a significant association would emerge when restricting our analysis to male perceivers rating female targets (as was reported in Brown & Sacco 2016a), but we did not find support for this (see Supplemental Materials). The association between extraversion preferences and germ aversion was not significant (with very strong evidence in favor of the null hypothesis), *b* = 0.07, *SE* = 0.08, *OR* = 1.08, 95% CI [0.93, 1.25], *p* = .33, *BF_01_* = 75.20 (see Figure 2b).

We did not find a significant association between extraversion preferences and individual differences in need to belong (with very strong evidence in favor of the null hypothesis), *b* = 0.08, *SE* = 0.07, *OR* = 1.08, 95% CI [0.92, 1.24], *p* = .30, *BF_01_* = 71.07 (see Figure 2c).

In the original study, it was predicted that people with a less restricted sociosexual orientation would show stronger extraversion preferences and that this association would be stronger for women rating men (vs. men rating women; Brown & Sacco 2016b). We therefore estimated a model in which face preference was regressed on sociosexual orientation (with higher scores indicating a less restricted sociosexual orientation), gender (–0.5 = men rating women, 0.5 = women rating men), and their interaction. There was a significant positive effect of sociosexual orientation, *b* = 0.42, *SE* = 0.14, *OR* = 1.51, 95% CI [1.19, 2.00], *p* = .002, *BF_10_* = 1.45. Participants with a less restricted sociosexual orientation showed stronger preferences for extraverted-looking individuals of the opposite sex. However, a Bayesian analysis only yielded anecdotal evidence in favor of this effect. The effect of gender was not significant (with strong evidence in favor of the null hypothesis), *b* = –0.77, *SE* = 0.40, *OR* = 0.46, 95% CI [0.21, 0.96], *p* = .05, *BF_01_* = 11.79. As predicted, there was a significant interaction effect between sociosexual orientation and gender, *b* = 0.55, *SE* = 0.27, *OR* = 1.74, 95% CI [1.06, 3.02], *p* = .04, *BF_01_* = 8.95 (see Figure 2d). However, Bayesian analyses yielded substantial evidence in favor of the null hypothesis and the observed pattern was *opposite* of what was found in the original study (Brown & Sacco 2016b). The positive association between a more unrestricted sociosexual orientation and extraversion preferences was stronger for men rating women (vs. women rating men).

#### Continuous ratings (10k faces)

**Figure 3:**
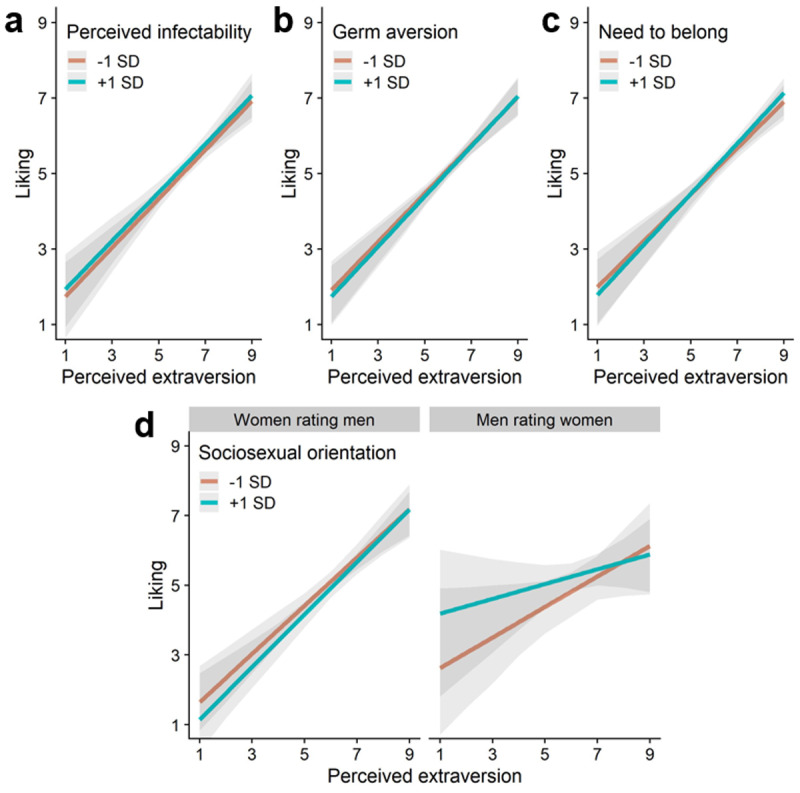
Predicted relation between facial extraversion and likeability ratings for participants scoring lower (–1 SD; orange lines) or higher (+1 SD; blue lines) on **(a)** perceived infectability, **(b)** germ aversion, **(c)** need to belong, **(d)** sociosexual orientation (the graph displays results for the predicted interaction effect with participants’ gender; Study 2).

Next, we analyzed participants’ ratings of the face stimuli taken from the 10k Faces Database. We examined associations between facial extraversion preferences and the four individual difference measures. Four separate multilevel linear regression models with separate random intercepts per participant and target were estimated. We regressed likeability ratings on apparent extraversion, each individual difference measure of interest, and their interaction (results for all models are displayed in Figure 3). The interaction effects represent the crucial tests of our hypotheses, as they show whether associations between targets’ apparent extraversion and participants’ likeability ratings were moderated by our variables of interest.

First, we examined whether participants scoring higher on pathogen concern showed weaker extraversion preferences. The interaction effect between apparent extraversion and perceived infectability was not significant (with decisive evidence in favor of the null hypothesis), *b* = –0.003, *SE* = 0.009, 95% CI [–0.02, 0.01], *p* = .78, *BF_01_* > 1000 (see Figure 3a), and neither was the interaction effect between apparent extraversion and germ aversion (with decisive evidence in favor of the null hypothesis), *b* = 0.01, *SE* = 0.01, 95% CI [–0.01, 0.03], *p* = .15, *BF_01_* > 1000 (see Figure 3b). We also tested whether a significant association for perceived infectability would emerge when restricting our analysis to male perceivers rating female targets (as was reported in Brown & Sacco 2016a), but we did not find support for this (see Supplemental Materials).

There was a significant interaction effect between apparent extraversion and need to belong, *b* = 0.02, *SE* = 0.01, 95% CI [0.01, 0.04], *p* = .01, *BF_01_* = 242.8 (see Figure 3c). However, this difference was small and a Bayesian analysis yielded decisive evidence in favor of the null hypothesis. For participants who scored at least one standard deviation higher than the mean on need to belong, a 1-point increase in apparent extraversion was associated with a 0.68-point increase in liking on our 9-point scale, *b* = 0.68, *SE* = 0.10, 95% CI [0.47, 0.82], *p* < .001, *BF_10_* > 1000. For participants who scored at least one standard deviation below the mean, on need to belong, a 1-point increase in apparent extraversion was associated with a 0.64-point increase in liking, *b* = 0.64, *SE* = 0.09, 95% CI [0.50, 0.86], *p* < .001, *BF_10_* > 1000.

We again examined whether participants with a less restricted sociosexual orientation would show stronger extraversion preferences and whether this association would be stronger for women rating men (vs. men rating women). We estimated a model in which likeability ratings were regressed on apparent extraversion, sociosexual orientation (with larger scores indicating a less restricted sociosexual orientation), gender (–0.5 = women rating men, 0.5 = men rating women), all two-way interactions, and the three-way interaction. The two-way interaction between apparent extraversion and sociosexual orientation was not significant (with decisive evidence in favor of the null hypothesis), *b* = –0.03, *SE* = 0.03, 95% CI [–0.09, 0.02], *p* = .28, *BF_01_* = 910.0, but the predicted three-way interaction with gender was, *b* = –0.12, *SE* = 0.06, 95% CI [–0.24, –0.01], *p* = .04, *BF_01_* = 99.12 (see Figure 3d). A Bayesian analysis yielded very strong evidence in favor of the null hypothesis.

We then analyzed the interaction between apparent extraversion and sociosexual orientation separately for male participants rating female targets and female participants rating male targets. As predicted, we found a non-significant interaction effect for male participants rating female targets (with very strong evidence in favor of the null hypothesis), *b* = –0.09, *SE* = 0.06, 95% CI [–0.21, 0.02], *p* = .12, *BF_01_* = 85.09, but a significant interaction effect for female participants rating male targets, *b* = 0.03, *SE* = 0.01, 95% CI [0.001, 0.06], *p* = .05, *BF_01_* = 446.1. In other words, and in line with the hypothesis, the results showed that female participants showed a greater liking for more extraverted-looking male targets and this association was more pronounced among more unrestricted (vs. more restricted) women. Note however, that this effect was small (see Figure 3d) and a Bayesian analysis showed decisive evidence in favor of the null hypothesis.

### Discussion

In Study 2, we examined the robustness of previous results by conducting replications that (a) used the same study design but a different set of face images and (b) used an alternative, arguably more robust method for measuring participants’ impressions and a third set of face images. In line with the results of our first study, we did not find consistent evidence for associations between facial extraversion preferences and individual differences in pathogen concern, need to belong, and sociosexual orientation (see Table 1 for an overview of which of the hypotheses from the original studies were supported).

## General Discussion

Even though recent work has shown that different perceivers reliably form different impressions of targets, it remains an open question which psychological characteristics of perceivers can explain these differences in social perception (Hehman et al. 2019). One series of studies tested if perceivers form more positive impressions of targets who appear more likely to address their needs, examining how various chronic motives (e.g., the need to belong) predict their likeability impressions of individuals varying in apparent extraversion (Brown & Sacco 2016b; Brown & Sacco 2016a; Brown & Sacco 2017). We outlined several critical limitations of this work and conducted two preregistered replication studies of three key findings.

Moving from a relatively close (Study 1, *n* = 273) to conceptual replications using two alternative stimulus sets and both the original and an improved study design (Study 2, *n* = 367), we tested whether participants with higher affiliative needs (Brown & Sacco 2017), lower pathogen concern (Brown & Sacco 2016a), and a less restricted sociosexual orientation (Brown & Sacco 2016b) form more positive impressions of extraverted-looking individuals. Across our different tests, we did not find clear support for any of the three hypotheses and Bayesian analyses yielded (usually strong) support for the null. These results emerged across two samples of participants, three stimulus sets, and two different study designs. Table 1 shows an overview of whether results of the frequentist and Bayesian analyses supported the predictions and results of the original studies.^6^

### Comparison and Integration with Previous Work

For each of the three primary hypotheses (regarding the role of individual differences in affiliative needs, pathogen concern, and sociosexual orientation), we find broadly consistent null results across our two participant samples and three stimulus sets. What can explain this difference in results compared to the original studies? The present results are based on considerably larger sample sizes (with regard to both participant and stimulus samples) than the original results, making it doubtful that our studies were underpowered to detect the original effects. We of course cannot rule out the possibility that the current studies were underpowered to detect a smaller, true effect. However, this seems less probable given that our Bayesian analyses consistently showed strong evidence in favor of the null hypothesis, rather than weak or anecdotal evidence, which we would expect with underpowered tests yielding non-diagnostic results.

Alternatively, the discrepant results may be due to differences between the studies (i.e., moderators that we did not model here). One salient difference is that the present studies were conducted during the COVID-19 pandemic. For example, it is plausible that the pandemic increased participants’ pathogen concern (due to the increased risk of infection and the salience of infectious disease) and affiliative needs (due to decreases social contact resulting from social distancing rules). However, it is less clear how this would lead to the observed null results. If there actually is a positive association between affiliative needs and extraversion preferences, then this association should still emerge if an exogenous factor, such as COVID-19, increased average scores. It is possible that the pandemic influenced some variable to such an extent that we did not obtain significant associations due to floor or ceiling effects, but our results speak against this (see Table S1 in the Supplemental Materials).

There are of course many other factors that differed between the current and the original studies. We recruited Dutch university students for our studies, whereas the original studies relied on samples of U. S. American participants recruited from Amazon Mechanical Turk. Thus, our participants were on average younger and more likely to be female. It is plausible that they were also more liberal and of higher socioeconomic status. While these differences are important to note, it is not clear how they could account for the different results observed here. The theoretical reasoning behind the original results would lead to similar predictions for the current studies. In the Supplemental Materials, we provide a more detailed overview of key differences between the original and replication studies (see Table S4). Additional studies would be needed to test whether one of these factors moderates the emergence of the tested associations.

In the current studies, we examined correlations between chronic social motives and impressions of individuals varying in perceived extraversion. An alternative way to test the role of social motives is by experimentally manipulating them (see, for example, Brown, Medlin et al. 2019; Brown, Sacco, et al. 2019; Brown & Sacco 2016a). Overall, experimental tests seem to converge more with the present null findings. Making disease threats salient did not affect participants’ extraversion preferences (Brown, Medlin et al. 2019; Brown & Sacco 2016a). In another study, increasing participants’ need to belong by socially excluding them increased extraversion preferences, but only for male participants (Brown, Sacco et al. 2019). Overall, considering both correlational and experimental approaches, the existing evidence casts doubt on the notion that affiliative needs or pathogen concern are related to preferences for extraverted-looking targets.

### Limitations and Future Directions

One limitation of the current studies is that the majority of our participants were female. Due to our relatively low number of male participants (Study 1: *n* = 81, Study 2: *n* = 56), the current studies may have been underpowered to detect potential sex differences for the association between sociosexual orientation and extraversion preferences (though Bayesian analyses indicated very strong evidence in favor of the null, which somewhat alleviate this concern).

Another limitation is the inherent difficulty of manipulating specific attributes of faces while keeping all others constant. Individuals who score high on perceived extraversion also score high on perceived attractiveness and other dimensions (Batres & Shiramizu 2022). Morphing the faces of low- and high-scoring individuals does not address this issue, as the resulting prototypes will not only differ on the target dimension, but also on other dimensions. While this is an inherent feature of people’s impressions (i.e., impression along different dimensions are correlated), it also means that it will remain somewhat ambiguous if, for example, people like an individual because they are perceived as scoring high on extraversion or because they are perceived as scoring high on a dimension correlated with perceived extraversion (e.g., attractiveness). Future studies should control for perceptions that (a) are known to be correlated with the target dimension and (b) are plausible confounds in a specific study context (e.g., people with an unrestricted sociosexual orientation might prefer extraverted-looking individuals but they should also prefer attractive-looking individuals).

We see two important avenues to improve the robustness of work on this topic. First, many studies, including the three that we tried to replicate here, relied on the same set of stimuli. To ensure that results are not due to idiosyncrasies of sampled stimuli, future studies should rely on other, larger, and more diverse stimulus sets. Second, the study design that was used in most previous research on this topic, in which impressions are measured by showing participants morphed version of the same face in a two-alternative forced-choice design, has been criticized for its poor external validity and tendency to produce false positive inferences (Bovet et al. 2022; DeBruine 2020). Serial rating tasks, such as the one employed in Study 2, in which participants view unmanipulated faces (with naturalistic variation in, for example, apparent extraversion) should produce more valid results.

The present studies should not be interpreted as providing evidence against the more general idea that perceiver characteristics influence how individuals with a certain appearance are judged. Variance decomposition analyses show that such target × perceiver effects explain a considerable amount of variance in first impressions (although they do not show which perceiver characteristics explain this; Hehman et al. 2019). The present studies simply show that evidence for the role of the specific perceiver characteristics that were proposed in prior work (Brown & Sacco 2016a; Brown & Sacco 2016b; Brown & Sacco 2017) is, at best, less robust and generalizable than previously thought. Given the methodological limitations of the original studies, the larger sample sizes and improved methods and analysis approach in the present studies, and the (in our judgment) absence of strong reasons for why the hypothesized associations should *not* be observed under the conditions that we tested them in, it is also possible that the original findings represent false positive results (in the Supplemental Materials, we provide an additional reason for this interpretation). While it can never be fully ruled out that unmodeled moderators explain the different results, the data collected in the present studies do not provide consistent evidence for the associations hypothesized in the original studies.

## Data Accessibility Statement

All data, analysis scripts, and preregistration documents for the current studies are available at the Open Science Framework (https://osf.io/eugd4). Our preregistrations specify the study design, the planned sample size and stopping rule for data collection, data exclusion criteria, and planned analyses. We explicitly mention any deviation from our preregistered approach. We report all manipulations, measures, and data exclusions.

## Additional File

The additional file for this article can be found as follows:

10.5334/irsp.996.s1 Supplementary Materials. Additional information and results.

## Footnotes

Although no gender differences were explicitly hypothesized, follow-up analyses that tested for an association between perceived infectability and extraversion preferences showed a significant effect for male participants rating female targets, a “marginally significant” effect for female participants rating male targets, and no significant effects for same-sex ratings.

This prediction was derived from work showing that extraverted men are more likely to exhibit several attributes that make them undesirable partners for women, such as increased infidelity (Nettle 2005). As this only imposes a cost on women in longer-term relationships, women with a more unrestricted sociosexual orientation, who are more focused on shorter-term relationships, are expected to show a stronger preference for extraverted men. Similar patterns were not expected for men judging women (for a more detailed explanation, see Brown & Sacco 2016b).

We also preregistered to test for a positive association between participants’ extraversion and their facial extraversion preferences (Sacco & Brown 2018). Due to a technical error, participants’ extraversion scores were not recorded and we were only able to test this hypothesis in Study 2.

In line with Brown and Sacco (2016b) and our preregistered analysis plan, we omitted 42 participants who were above the age of 40 or did not identify as heterosexual from analyses examining associations between sociosexual orientation and extraversion preferences.

In line with Brown and Sacco (2016b) and our preregistered analysis plan, we omitted 78 participants who were above the age of 40 or did not identify as heterosexual from analyses examining associations between sociosexual orientation and extraversion preferences.

We did not include the results on perceiver extraversion, which showed a consistently positive association in Study 2 across both analysis types, in the table because we considered this hypothesis to be of secondary importance (as the underlying process that might explain this association does invoke perceivers’ chronic needs, which was the primary focus of the original studies and the present studies).
