# Supplementary material for: Who Likes Extraverts? Testing the Interplay Between Perceiver Needs and Target Appearance in Impression Formation
Source: Int Rev Soc Psychol. 2026 Mar 26;39:2. doi: 10.5334/irsp.996 (PMC13025157; doi:10.5334/irsp.996)
Supplement: Supplementary Materials. — Additional information and results. [file irsp-39-996-s1.pdf]

Supplemental Materials

Here, we report additional analyses that were omitted from the main text for the sake of brevity.

Power Analyses

The main goal of Study 1 was to examine associations between likeability ratings of individuals varying in perceived extraversion and various individual difference measures. An a priori power analysis showed that a sample of 193 participants is needed to detect a small-to-medium correlation (r = .2) with 80% power (and α = 5%). We therefore aimed to recruit a minimum sample of 193 participants with the final sample being determined by how many participants completed the study within three weeks. Also note that we conducted Bayesian analyses, which can be used to quantify evidence in favor of the null and alternative hypothesis. This can give insights into whether non-significant results represent non-diagnostic evidence because of low power or diagnostic evidence in favor of a null effect.

As in Study 1, the main goal of Study 2 was to examine associations between likeability ratings of individuals varying in perceived extraversion and various individual difference measures. We increased our planned sample size compared to Study 1, planning for 95% (vs. 80%) power. An a priori power analysis showed that a sample of 319 participants is needed to detect a small-to-medium correlation (r = .2) with 95% power (and α = 5%). We therefore aimed to recruit a minimum sample of 319 participants with the final sample being determined by how many participants completed the study within three weeks. Note that this is only an approximation as our analyses focus on the results of multilevel regression models.

The final sample sizes of 273 and 367 participants in Studies 1 and 2, respectively, were somewhat larger than the samples in the original studies (n = 155 in Brown & Sacco, 2016a; n = 207 in Brown & Sacco, 2016b; n = 149 in Brown & Sacco, 2017). In the original studies, not all associations that we aimed to examine here were reported as standardized effect sizes. For sociosexual orientation, an association was specifically hypothesized for female perceivers rating male targets. However, in the original study, only the association for male faces irrespective of perceivers' gender was reported (r = .21; Brown & Sacco, 2016b). Study 1 had 94% power to detect an effect of this size, whereas Study 2 had 98% power. For the need to belong, an effect size of r = .16 was reported in the original study (Brown & Sacco, 2017). Study 1 only had 76% power to detect an effect of this size, and Study 2 had 87% power. For pathogen concern, the

relevant effect sizes were not reported in the original study (Brown & Sacco, 2016a). Note that these are only conservative approximations as our multilevel regression analyses should be more powerful in detecting the hypothesized associations (Baayen et al., 2008).

Bayesian Analyses

We conducted Bayesian analyses following the approach outlined in Wagenmakers (2007). For all critical tests, we compute an approximation of the Bayes factor and we examined to what extent it supported the null vs. alternative hypothesis. We first estimated regression models with and without the key variable of interest (see the main manuscript). For example, to test the association between extraversion preferences and the need to belong, we estimated a generalized linear multilevel regression model with random intercepts per participant and target, in which we regressed extraversion preferences (0 = introverted-looking face preferred, 1 = extraverted-looking face preferred) on participants' need to belong scores. This constituted our focal model. To construct an associated null model, we again estimated the same model, but omitted the focal variable we aimed to test (here, need to belong). Next, we computed the model fit for both the focal and the null model using the Bayesian information criterion (BIC). Bayes factor approximations were computed using the BIC estimates with the following formula: $BF_{10} \approx exp\left(\frac{BIC(H_0) - BIC(H_1)}{2}\right)$, where BF_{10} represents the Bayes factor in favor of the alternative hypothesis and $BIC(H_1)$ and $BIC(H_0)$ represent the fit of the models with and without the variable of interests (Wagenmakers, 2007). These analysis steps can be reproduced with our provided code (https://osf.io/eugd4/?view_only=714b82f50c3147b586e75eb8296ce6a7).

Descriptive Statistics

Table S1

Descriptive statistics for Studies 1 and 2

	Study 1			Study 2		
	α	*M*	*SD*	*α*	*M*	*SD*
Male (%)	-	29.67	-	-	15.26	-
Age	-	23.18	6.14	-	19.90	3.70
Extraversion preference (%)	-	49.45	13.75	-	83.07	15.80
Perceived infectability	.86	3.36	1.16	.87	3.24	1.17
Germ aversion	.70	4.07	1.01	.74	3.76	1.07
Need to belong	.75	3.22	0.58	.75	3.25	0.59
Sociosexual orientation	.83	3.61	1.39	.84	3.38	1.35
Extraversion	-	-	-	.82	3.27	0.69

Note. The mean for “male” denotes the percentage of male participants in the sample. The mean for “extraversion preference” denotes the percentage of times participants chose the extraverted face.

Table S2

Correlations between individual difference variables in Studies 1 and 2

	Correlation				
Measure	1	2	3	4	5
1. Perceived infectability	—	.16**	.11*	-.01	-.05
2. Germ aversion	.33***	—	.03	-.25***	-.01
3. Need to belong	.18**	.01	—	-.05	-.17**
4. Sociosexual orientation	-.05	-.16**	.04	—	.22***
5. Extraversion	—	—	—	—	—

Note. Correlations below the diagonal show results of Study 1; correlations above the diagonal show results of Study 2.

*$p < .05$, **$p < .01$, ***$p < .001$

Figure S1

Distributions of individual difference scores in Study 1

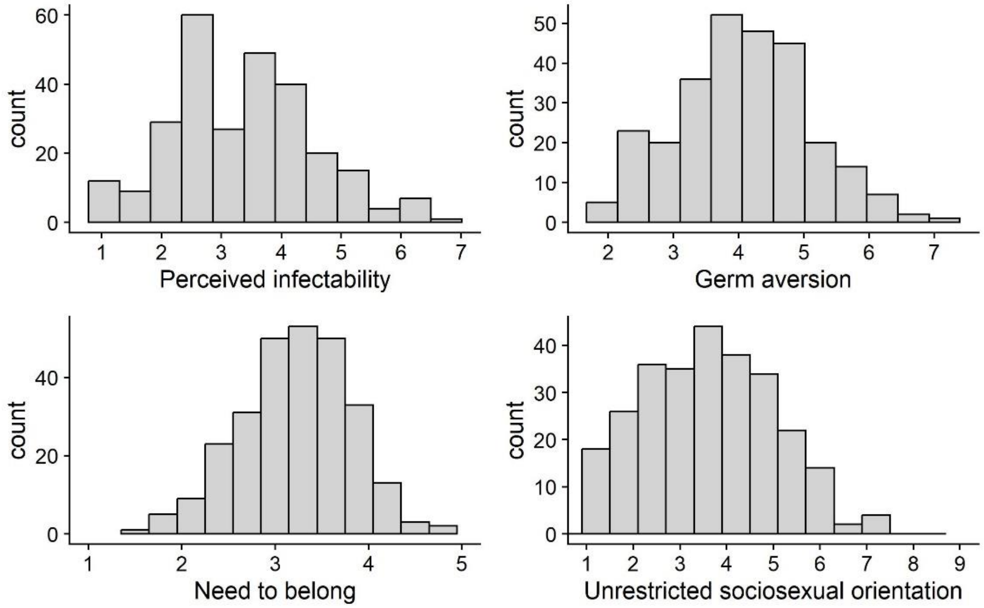

Figure S2

Distributions of individual difference scores in Study 2

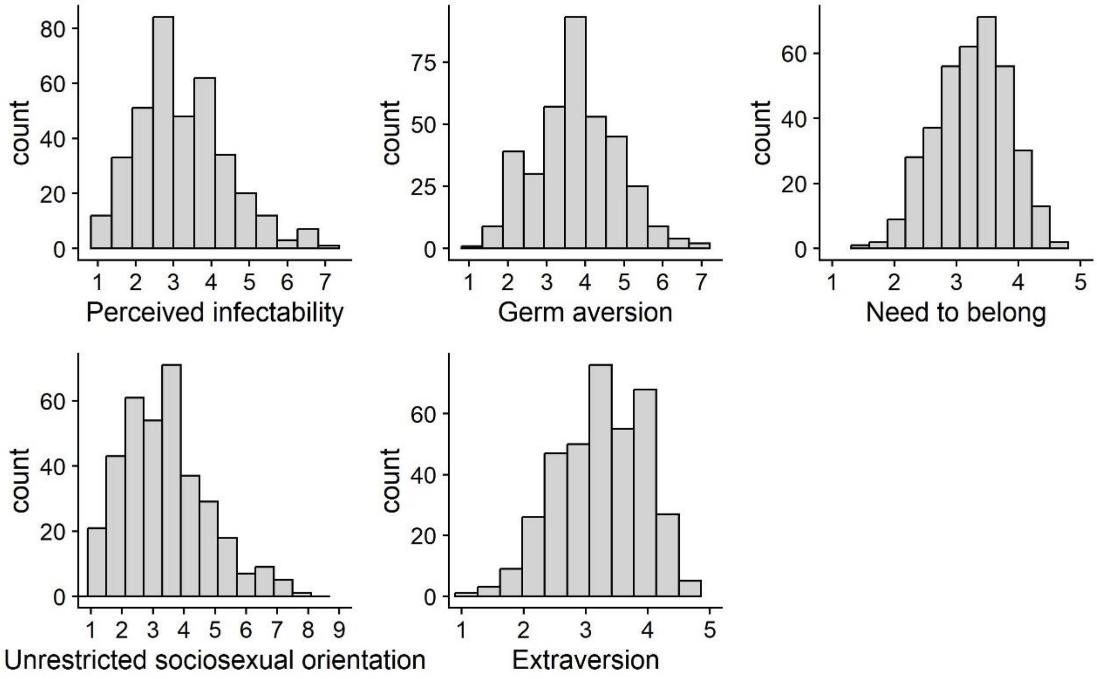

Liking Extraverted Looks

In both studies, we also explored if people showed a preference for extraverted-looking targets in general. In Study 1, we examined this by testing whether extraversion preferences were stronger than expected by chance (i.e., 50%) by examining the intercept in a multilevel logistic regression model with separate random intercepts per participant and target. The intercept was not significant and a Bayesian analysis indicated decisive evidence in favor of the null hypothesis, b = -0.02, SE = 0.11, OR = 0.98, 95% CI [0.79, 1.22], p = .87, BF_{01} = 103.2. Participants chose the extraverted-looking face 49.45% of the time. Thus, in contrast to earlier studies with the same stimuli (Brown & Sacco, 2016b, 2016a, 2017), participants did not show a preference for extraverted-looking or introverted-looking faces. In line with these earlier studies, we did find that extraversion preferences differed for male and female targets. Adding target gender to the model (0 = female target, 1 = male target) yielded a significant effect with decisive evidence in favor of the alternative hypothesis, b = -0.86, SE = 0.16, OR = 0.42, 95% CI [0.31, 0.58], p < .001, BF_{10} = 722.9. When rating male faces, participants chose the extraverted-looking face 39.96% of the time, but when rating female faces, participants chose the extraverted-looking face 58.94% of the time.

In Study 2, when analyzing ratings of targets from the Basel Face Database, we found that participants chose the extraverted-looking face 83.07% of the time. We tested whether this preference was stronger than expected by chance (i.e., 50%) by examining the intercept in a multilevel logistic regression model with separate random intercepts per participant and target. This yielded a significant intercept and decisive evidence in favor of the alternative hypothesis, b = 2.22, SE = 0.15, OR = 9.21, 95% CI [7.06, 12.03], p < .001, BF_{10} > 1000. Thus, participants generally preferred extraverted-looking faces. We also tested if this preference was different for male and female targets. Adding target gender to the model (0 = female target, 1 = male target) did not yield a significant effect with decisive evidence in favor of the null hypothesis, b = 0.04, SE = 0.26, OR = 1.04, 95% CI [0.62, 1.67], p = .89, BF_{01} = 120.0.

To test whether participants had more positive impressions of more extraverted-looking targets from the 10k Faces Database, we regressed likeability ratings on the apparent extraversion of targets in a multilevel linear regression model with separate random intercepts per participant and target. This yielded a significant positive effect and decisive evidence in favor of the alternative hypothesis, b = 0.65, SE = 0.09, 95% CI [0.50, 0.81], p < .001, BF_{10} >

1000. Thus, participants generally rated more extraverted-looking targets as more likeable. We again tested whether the gender of targets moderates this effect. Adding target gender to the model (0 = female target, 1 = male target) did not yield a significant interaction effect between target gender and apparent extraversion with decisive evidence in favor of the null hypothesis, $b = 0.21$, $SE = 0.21$, 95% CI [-0.21, 0.62], $p = .32$, $BF_{01} = 221.7$.

The Role of Perceivers' Extraversion

In Study 2, we also measured perceivers' extraversion to test if it relates to their facial extraversion preferences. When analyzing impressions of targets from the Basel Face Database, we found a positive effect of participants' extraversion on their facial extraversion preferences (with decisive evidence in favor of the alternative hypothesis), $b = 0.36$, $SE = 0.07$, $OR = 1.43$, 95% CI [1.25, 1.66], $p < .001$, $BF_{10} > 1000$ (see Figure S3a). A one standard deviation increase in extraversion was associated with a 2.69 percentage point increase in extraversion preferences. In other words, more extraverted participants showed more positive impressions of extraverted-looking targets.

When analyzing impressions of targets from the 10k Face Database, we found a significant interaction effect between apparent extraversion and participants' level of extraversion (with decisive evidence in favor of the alternative hypothesis), $b = 0.05$, $SE = 0.01$, 95% CI [0.04, 0.07], $p < .001$, $BF_{10} > 1000$ (see Figure S3b). Participants who scored at least one standard deviation higher than the mean on extraversion showed a stronger liking for extraverted-looking individuals, $b = 0.74$, $SE = 0.10$, 95% CI [0.46, 0.80], $p < .001$, $BF_{01} > 1000$, than participants who scored at least one standard deviation below the mean on extraversion, $b = 0.63$, $SE = 0.09$, 95% CI [0.51, 0.96], $p < .001$, $BF_{10} > 1000$. Although the size of the interaction effect was relatively small, it was more than twice as strong as the interaction effect for need to belong.

Figure 3

Predicted relation between (a) facial extraversion preferences (Basel Faces stimuli) and perceivers' extraversion, and (b) between facial extraversion and likeability ratings (10k Faces stimuli) for perceivers scoring lower (-1 SD; orange lines) or higher (+1 SD; blue lines) on extraversion.

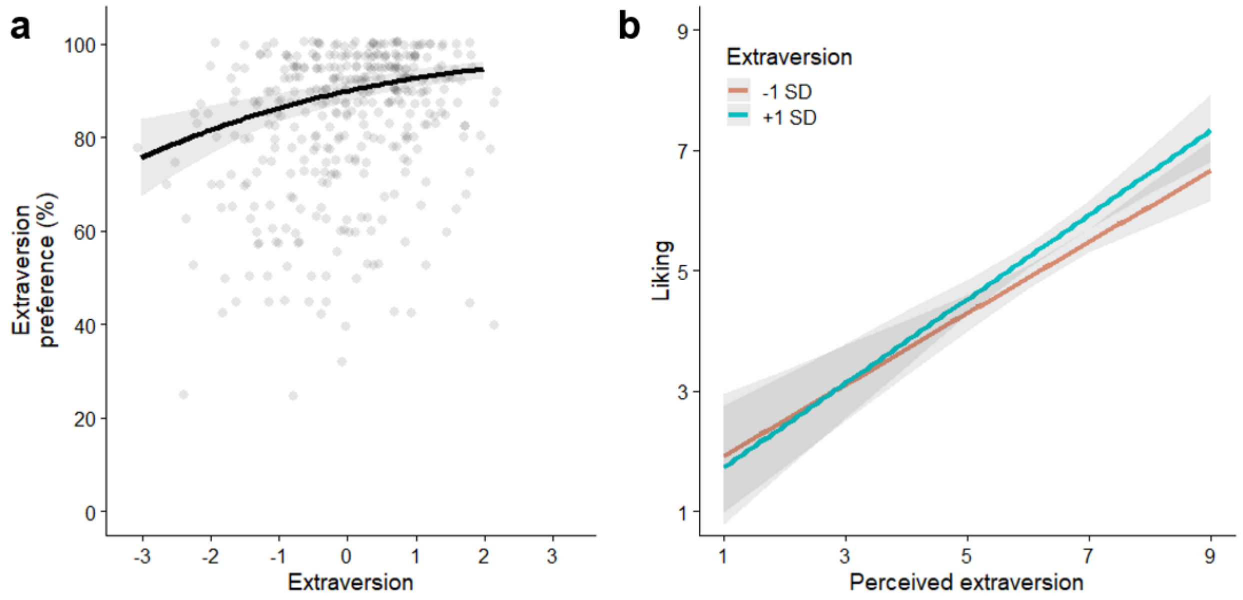

Correlational analyses

Table S3 shows correlation between all measured perceiver differences and their extraversion preferences measured with the same stimuli as the original studies in Study 1 and with the Basel face stimuli in Study 2. The results were broadly in line with the results we report in the main manuscript (see Table 1), with one notable exception: We found a significant association for perceived infectability using multilevel regression analyses, but not for the correlational analysis of averaged extraversion preference scores.

Table S3

Correlations between perceiver difference variables and the percentage of times perceivers preferred the extraverted-looking target in Study 1 (original stimuli) and Study 2 (Basel Faces stimuli).

	Study 1	Study 2
Perceived infectability	-.003	-.10
Germ aversion	-.06	.03
Need to belong	-.09	.07
Sociosexual orientation (women rating men)	-.03	.07
Sociosexual orientation (men rating women)	-.01	.17
Extraversion	—	.25***

*$p < .05$, **$p < .01$, ***$p < .001$

Perceived Infectability: Subgroup Analyses

Brown and Sacco (2016a) hypothesized that participants with increased pathogen concern would show decreased preferences for extraverted-looking others. Although they only found a marginally significant association for the perceived infectability subscale (and no significant association for the germ aversion subscale), they did find a significant association for male participants rating female faces. We examined the robustness of this finding as this pattern was (a) not predicted a priori, (b) just below the significance threshold, and (c) the three-way interaction between perceived infectability, participant gender, and target gender was only marginally significant.

In Study 1, there was no significant association between perceived infectability and extraversion preferences for male participants rating female faces (with very strong evidence in favor of the null hypothesis), $b = 0.03$, $SE = 0.09$, $OR = 1.03$, 95% CI [0.86, 1.23], $p = .74$, $BF_{01} = 38.05$. In Study 2, when analyzing participants' preferences for the Basel faces, there was no significant association between perceived infectability and extraversion preferences for male participants rating female faces (with strong evidence in favor of the null hypothesis), $b = -0.25$, $SE = 0.24$, $OR = 0.78$, 95% CI [0.47, 1.27], $p = .30$, $BF_{01} = 20.55$. Finally, when analyzing participants' ratings of the 10k faces, the interaction between apparent extraversion and perceived infectability was not significant for male participants rating female faces (with decisive evidence in favor of the null hypothesis), $b = 0.04$, $SE = 0.06$, 95% CI [-0.07, 0.15], $p =$

.47, BF_{01} = 270.0. Thus, we did not find evidence that men who score high on perceived infectability show stronger preferences for extraverted-looking women.

Differences Between the Original and the Replication Studies

Here, we provide a more detailed overview of the similarities and differences between the original studies and the replication studies. We focus on nine dimensions, similar to the ones identified in Brandt and colleagues' (2014) "replication recipe". The three key associations that we attempted to replicate here associations for pathogen concern, the need to belong, and sociosexual orientation) were originally tested in different studies, with slightly different methodologies, and published in different papers (Brown & Sacco, 2016b, 2016a, 2017). To keep the comparison concise, we provide a general classification of the differences in Table S4 and we describe important similarities and differences.

Table S4

Overview of key similarities and differences between the original and replication studies

	Study 1 (original stimuli)	Study 2 (Basel face stimuli)	Study 2 (10k face stimuli)
Participant population	Different	Different	Different
Remuneration	Different	Different	Different
Location	Exact	Exact	Exact
Instructions/ procedure	Close	Close	Close
Stimulus set	Exact	Different	Different
Stimulus presentation	Exact	Exact	Different
Face rating measure	Exact	Exact	Different
Individual difference measures	Exact	Exact	Exact
Analysis approach	Different[a]	Different[a]	Different[a]

[a] We report results from the analysis approach that was used in the original studies in the Supplemental Materials.

Participant Population, Remuneration, and Study Location

The original studies sampled U.S. participants via Amazon Mechanical Turk. Participants were paid $0.35. The average age of in the three original samples varied between 30 and 40 years with considerable variability (*SD*s ranged from 6 to 14), as is common for MTurk samples. In Study 1, we recruited participants via several Facebook groups of Dutch universities and participation was voluntary. The sample was younger with less age variability (M_{age} = 23.18 years, SD_{age} = 6.14). In Study 2, first-year psychology students from a Dutch university who completed the study in return for partial course credit. The sample was again younger with less age variability (M_{age} = 19.90 years, SD_{age} = 3.70). The replication sample sizes (237 and 367) were larger than the original sample sizes (149 for testing the need to belong, 150 for testing pathogen concern, 187 for testing sociosexual orientation). The original studies and the replication studies were completed online and in English.

Instructions, Procedure, and Measures

The original authors shared the stimuli that were used in their studies and we used the same stimuli in Study 1. In Study 2, we used two alternative stimulus sets to test the generalizability of the results. In the original studies, a series of face pairs differing in apparent extraversion were shown next to each and participants selected which face they prefer. We used the same presentation method and measure of facial extraversion preferences in Study 1 and in Study 2 (for the Basel Faces stimuli). For our alternative test with the 10k Faces stimuli in Study 2, we used a different presentation method (showing images one at a time) and a different measure of facial extraversion preferences (participants indicated their liking on a 9-point Likert scale). We measured individual differences in pathogen concern, the need to belong, and sociosexual orientation with the same scales that were used in the original studies. Based on the description of the original studies' procedure, we only identified one additional potentially important difference. In the original study that tested the role of pathogen concern, participants were also exposed to slide show of images that were meant to make disease accessible to participants in the experimental condition, or images that were meant to evoke negative affect in the control condition. However, this manipulation did not affect the outcome variable and no interactions with trait pathogen concern were detected.

Analysis Approach

In the original studies, the predicted associations were tested with correlation coefficients (Brown & Sacco, 2017) or multiple regression models (when testing for interactions with perceiver and target sex Brown & Sacco, 2016a, 2016b). For both analyses, average preferences for extraverted-looking individuals were first computed for each participant by calculating the percentage of trials on which they preferred the more extraverted-looking face. These averages were then related to the individual difference variables. For our replication studies, we report correlation coefficients for all associations in the Supplemental Materials. However, our primary analyses use multilevel regression models that are better suited for modeling the nested structure of the data, where individual ratings are nested within participants and stimuli.

Potential Influences on the Results

In the main manuscript, we discuss key differences in more detail, including how likely it is that each could have caused the differences in observed results between the original and replication studies. It is of course possible that any of the differences listed here, or any other more specific difference that resulted from recruiting different people from different countries at a different point in time, could explain why significant associations were observed in the original studies but not in our replication studies. However, before such a conclusion is drawn, these predictions should be tested in future work. Given the methodological limitations that were identified in the original studies, the larger sample sizes and improved methods and analysis approach in the replication studies, and the fact that we have not identified strong reasons for why the hypothesized associations should *not* be observed under the conditions that we tested them in the present replication studies, it is also possible that the original findings represent false positive results. Thus, at present, the only cautious conclusion that can be drawn is that the associations hypothesized in the original studies are less generalizable and robust than previously thought, and that additional data (ideally from preregistered studies with improved designs) would be needed to support the original claims.

An Additional Issue: Correcting for Multiple Testing

Here, we highlight an additional criticism of the original studies: It is possible that the original findings represent false positives resulting from testing the same hypothesis multiple times without the necessary corrections for multiple testing. Error correction fundamentally tries to avoid drawing false conclusions based on the data (see, for example, Lakens, 2016; Rubin,

2024). If the same hypothesis is tested four times (e.g., examining the same association for male and female perceivers and targets), whether or not the alpha threshold should be adjusted partly depends on which conclusion a researcher aims to draw. For example, if a researcher only finds an association for male perceivers rating female targets, but concludes that the association exists in general (i.e., that a more general hypothesis was supported), then this is clearly problematic because the researcher had four chances of finding support for the hypothesis (Simmons et al., 2011). We believe that this is one of the clearer cases in which error correction is required (Lakens, 2016; Rubin, 2024).

These problematic inferences, drawing more general conclusions even though only one or a few sub-tests showed support for the hypothesis, are sometimes made in the original studies. For example, in Brown and Sacco (2016a), the authors conclude in the General Discussion that they “provide preliminary evidence for how BIS activation influences face perception in the context of facial cues associated with extraversion, albeit only in terms of dispositional activation of pathogen concern” (p. 283) and that they “demonstrate motivation-influenced preference shifts for extraverted personality traits, as communicated by facial cues” (p. 283). These general inferences are inappropriate given that they seemingly conducted eight tests (two subscales × two perceiver genders × 2 target genders) and only one of these yielded a significant result.

Similarly, in Brown and Sacco (2016b), an association for sociosexual orientation is specifically hypothesized for female (but not male) perceivers rating male (but not female) targets. An association was found for male targets, but this was not moderated by perceiver sex, as predicted. This was not described clearly in the General Discussion, where a more positive conclusion was drawn that “sociosexually unrestricted women are thus more willing to make this trade-off such that their preferences for more extraverted men reflects […]” (p. 126). Some of these unexpected results—the moderators observed in the studies were not predicted a priori based on the reviewed theory— may point to interesting boundary conditions. However, they may also constitute false positives as increasing the number of statistical tests (e.g., by analyzing different subgroups of participants and targets) without adjusting the significance threshold is bound to produce significant results (Simmons et al., 2011).

References

Brown, M., & Sacco, D. F. (2016a). Avoiding extraverts: Pathogen concern downregulates preferences for extraverted faces. *Evolutionary Psychological Science*, *2*(4), 278–286. https://doi.org/10.1007/s40806-016-0064-6

Brown, M., & Sacco, D. F. (2016b). Unrestricted sociosexuality predicts preferences for extraverted male faces. *Personality and Individual Differences*, *108*, 123–127. https://doi.org/10.1007/978-3-319-12616-6

Brown, M., & Sacco, D. F. (2017). Greater need to belong predicts a stronger preference for extraverted faces. *Personality and Individual Differences*, *104*, 220–223. https://doi.org/10.1016/j.paid.2016.08.012

Lakens, D. (2016). Why you don't need to adjust your alpha level for all tests you'll do in your lifetime. *The 20% Statistician*. https://daniellakens.blogspot.com/2016/02/why-you-dont-need-to-adjust-you-alpha.html

Rubin, M. (2024). Inconsistent multiple testing corrections: The fallacy of using family-based error rates to make inferences about individual hypotheses. *Methods in Psychology*, *10*, 100140. https://doi.org/10.1016/j.metip.2024.100140

Simmons, J. P., Nelson, L. D., & Simonsohn, U. (2011). False-positive psychology: Undisclosed flexibility in data collection and analysis allows presenting anything as significant. *Psychological Science*, *22*(11), 1359–1366. https://doi.org/10.1177/0956797611417632

Wagenmakers, E.-J. (2007). A practical solution to the pervasive problems of p values. *Psychonomic Bulletin & Review*, *14*(5), 779–804. https://doi.org/10.3758/BF03194105
